# Diagnostic Value of Electroencephalography with Ten Electrodes in Critically Ill Patients

**DOI:** 10.1007/s12028-019-00911-4

**Published:** 2020-02-07

**Authors:** M. Brandon Westover, Kapil Gururangan, Matthew S. Markert, Benjamin N. Blond, Saien Lai, Shawna Benard, Stephan Bickel, Lawrence J. Hirsch, Josef Parvizi

**Affiliations:** 1 Department of Neurology, Massachusetts General Hospital, Boston, MA USA; 2 The Mount Sinai Hospital, New York, NY USA; 3 Stanford University Medical Center, Stanford, CA USA; 4 Stony Brook University Hospital, Stony Brook, NY USA; 5 Kaiser Permanente Medical Center, Panorama City, CA USA; 6 Keck Hospital of University of Southern California, Los Angeles, CA USA; 7 Zucker School of Medicine at Hofstra/Northwell, Long Island, NY USA; 8 Yale-New Haven Hospital, New Haven, CT USA

**Keywords:** Electroencephalography (EEG), Abbreviated EEG, Continuous EEG monitoring, Seizure prediction, Non-convulsive status epilepticus, Neuroemergencies

## Abstract

**Background:**

In critical care settings, electroencephalography (EEG) with reduced number of electrodes (reduced montage EEG, *rm*-*EEG*) might be a timely alternative to the conventional full montage EEG (*fm*-*EEG*). However, past studies have reported variable accuracies for detecting seizures using rm-EEG. We hypothesized that the past studies did not distinguish between differences in sensitivity from differences in classification of EEG patterns by different readers. The goal of the present study was to revisit the diagnostic value of rm-EEG when confounding issues are accounted for.

**Methods:**

We retrospectively collected 212 adult EEGs recorded at Massachusetts General Hospital and reviewed by two epileptologists with access to clinical, trending, and video information. In Phase I of the study, we re-configured the first 4 h of the EEGs in lateral circumferential montage with ten electrodes and asked *new* readers to interpret the EEGs without access to any other ancillary information. We compared their rating to the reading of hospital clinicians with access to ancillary information. In Phase II, we measured the accuracy of the same raters reading representative samples of the discordant EEGs in full and reduced configurations presented randomly by comparing their performance to majority consensus as the gold standard.

**Results:**

Of the 95 EEGs without seizures in the selected fm-EEG, readers of rm-EEG identified 92 cases (97%) as having no seizure activity. Of 117 EEGs with “seizures” identified in the selected fm-EEG, none of the cases was labeled as normal on rm-EEG. Readers of rm-EEG reported pathological activity in 100% of cases, but labeled them as seizures (*N* = 77), rhythmic or periodic patterns (*N* = 24), epileptiform spikes (*N* = 7), or burst suppression (*N* = 6). When the same raters read representative epochs of the discordant EEG cases (*N* = 43) in both fm-EEG and rm-EEG configurations, we found high concordance (95%) and intra-rater agreement (93%) between fm-EEG and rm-EEG diagnoses.

**Conclusions:**

Reduced EEG with ten electrodes in circumferential configuration preserves key features of the traditional EEG system. Discrepancies between rm-EEG and fm-EEG as reported in some of the past studies can be in part due to methodological factors such as choice of gold standard diagnosis, asymmetric access to ancillary clinical information, and inter-rater variability rather than detection failure of rm-EEG as a result of electrode reduction per se.

## Introduction

In 1958, a committee of the International Federation reported on a standardized method of recording and displaying electroencephalography (EEG) data, known as the International 10-20 system, to facilitate reliable interpretation of data acquired across different sites and readers [1]. Since then, we have continued to use the same EEG recording approach for widely different applications. The conventional 10-20 system—hereafter called *full montage* EEG (fm-EEG)—is used both in diagnosing epilepsy through detecting spikes and other epileptiform discharges, and in detecting status epilepticus in critically ill patients. In recent decades, increased demand for EEG monitoring in critically ill patients has been tempered by a shortage of trained EEG technologists and prohibitive costs of EEG monitoring in smaller medical centers, and subsequently, providing traditional EEG services has become increasingly difficult, especially after hours. Economic incentives to continue the status quo may be diminishing with the development of new current procedural terminology codes and proposed reimbursement cuts for long-term recordings using the traditional EEG approach.

An alternative approach is to use EEG with a reduced number of electrodes—hereafter called *reduced montage* EEG (rm-EEG)—to make EEG acquisition less dependent on specialized EEG technologists and to reduce EEG setup time. However, a major barrier to adoption of rm-EEG is the lack of systematic studies on the diagnostic utility of this method. While the use of rm-EEG recordings has been explored by a number of investigators, evaluation of the performance of rm-EEG in several of these studies has been limited in at least three domains: variability in reduced EEG design, asymmetric comparison between full and reduced EEGs, and selection of patterns of interest [2, 3].

### Confounding Factors in Prior Research

Prior studies on the diagnostic accuracy of abbreviated EEG have used rm-EEG arrays that vary both in total number of electrodes and in montage configuration [4–17]. These arrays intrinsically differ in their sensitivity to detect clinically significant patterns, and it is unwarranted to generalize findings across all forms of rm-EEG.

Many prior studies comparing abbreviated EEG systems to the 10-20 system have relied on a problematic “gold standard,” namely the original clinical EEG report written during the course of patient care. These reports are often the products of a critical discussion between a fellow and attending physician with access to several days of EEG data and additional clinical information (e.g., video, trending, and patient history). By comparison, studies of abbreviated EEG typically presented individual reviewers, often not the same epileptologists who originally reviewed the EEGs during patient care, with shorter, representative rm-EEG samples absent equivalent clinical information. These experimental settings differ in access to ancillary information and the EEG review process itself (consensus versus individual). EEG is already subject to substantial inter-rater variability, and asymmetric access to clinical information exaggerates the bias introduced by this variability [18–20]. Although consensus guidelines [21] have established criteria for classifying pathological, but non-ictal, EEG activity, such as rhythmic and periodic patterns, these can be difficult to distinguish from ictal activity in critically ill patients without full access to clinical information [22–25].

Overall, these three important problems contribute to the *classification problem* that results in differences in the naming and classification of the visualized EEG patterns, and therefore, discrepancies between fm-EEG and rm-EEG diagnoses observed in prior studies may not be attributable solely to a *detection problem*, i.e., lesser sensitivity due to electrode reduction [3].

Another important consideration in the study of the diagnostic value of rm-EEG is the selection of specific patterns whose detection would guide clinical management. Firstly, “epileptic abnormalities” can range from single epileptiform spikes to generalized status epilepticus, and the lower sensitivity of rm-EEG for detecting isolated spikes does not necessarily imply a lower sensitivity for detecting gross abnormalities. Secondly, although prior studies have unanimously and appropriately regarded electrographic seizures as the most valuable diagnostic abnormality, few have addressed the utility of rm-EEG for the detection of other pathological patterns that might help identify patients at higher risk of seizures. The dominant risk factor in early EEG for subsequent seizures is the presence of epileptiform abnormalities, particularly periodic discharges [26–29]. However, no prior studies have shown whether the sensitivity of early rm-EEG for these abnormalities is adequate to preserve this predictive value.

### Study Aims

The current study was designed to address two aims: (1) to replicate the decrement in accuracy of rm-EEG shown in prior studies, and (2) to discern whether the prior reports of a significant discordance between rm-EEG and fm-EEG diagnoses can be explained by methodological issues rather than detection failure as a result of electrode reduction per se. These aims were addressed in Phase I and Phase II of the study, respectively.

## Methods

### Overview of the Study Hypotheses and Study Design

In Phase I of the study, we compared the utility of fm-EEG and rm-EEG using similar methodology reported in several prior studies. Here, we defined the gold standard as the diagnostic impressions of clinical epileptologists reading fm-EEGs with full access to ancillary information (e.g., clinical, trending, and video data as well as prior labeling of the EEGs by technologists) and then asked new EEG raters to read the rm-EEG iterations of the same EEGs without access to ancillary information. We expected to find a significant discordance between rm-EEG and fm-EEG interpretations, especially in terms of diagnosing seizures.

In Phase II, after confirming discrepancies between rm-EEG and fm-EEG in the first phase of the study, we tested whether discrepancies between rm-EEG and fm-EEG could be attributed to the impact of ancillary (non-EEG) data and inter-rater variability rather than the reduction in the number of electrodes per se. We expected to find that discrepancies between fm-EEG and rm-EEG diagnoses would be largely reduced using a different gold standard and the same readers reading fm-EEG and rm-EEGs with symmetric access to information.

### Full and Reduced EEG Configurations

**Fig. 1:**
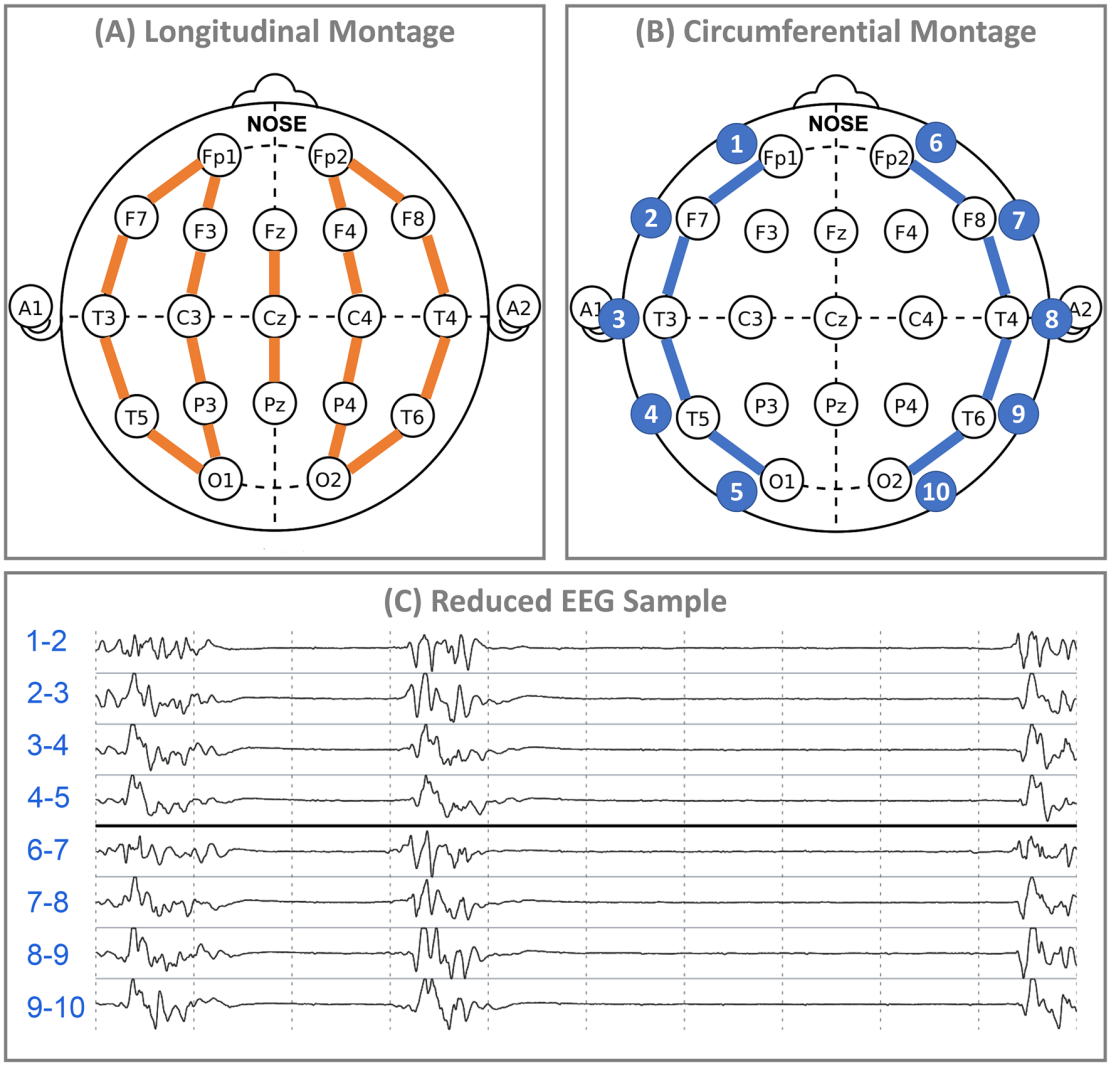
Full montage and reduced montage electroencephalogram construction. Electrodes used to construct bipolar anterior-posterior montages for conventional full montage (**A**) and reduced montage (**B**) electroencephalography (EEG), referred to as fm-EEG and rm-EEG, respectively. A sample of rm-EEG (**C**) showing burst suppression activity is shown, indicating the electrodes used to construct the montage. Readers could adjust display epoch time, scale, and high- and low-pass filters while reviewing EEGs in Phase I, but were not allowed to re-montage the EEG. Readers could not adjust any display settings in Phase II

We utilized the standard International 10-20 EEG system to define fm-EEG (Fig. 1A), which provides coverage of lateral, parasagittal, and midline regions. For rm-EEG, we focused on a configuration with ten electrodes (eight channels) covering the lateral circumference of the scalp (Fig. 1B). The rationale for the choice of this rm-EEG configuration was as follows. First, a new medical device recently developed by *Ceribell* (see conflict of interest) uses this configuration, and since the device is in clinical use in some of the authors’ institutions, findings of our study will help us adjust our own views of its accuracy. Second, a recent report by two of the co-authors (JP and KG) reported that seizures limited to midline and parasagittal regions are rare (occurring in less than 1% of all EEG recordings) [30, 31]. Third, this rm-EEG configuration has been previously demonstrated to have high sensitivity and specificity for generalized or hemispheric seizures and high specificity for generalized or hemispheric rhythmic or periodic patterns that are frequently seen in critically ill patients [32], in which case the lateral circumferential rm-EEG configuration should incur only minimal loss in diagnostic sensitivity.

### Study Population

Adult patients (> 18 years old) who underwent continuous EEG monitoring at Massachusetts General Hospital (MGH) between August 1, 2010, and June 2, 2012, were retrospectively identified with the approval of the Partners Human Research Committee (Institutional Review Board) [26, 27]. In prior studies, one of the authors (MBW) reported this cohort of 625 EEGs as containing 168 EEGs with seizures (135 with seizures within 4 h and 33 with seizures after 4 h) and 457 without seizures, including both EEGs with and without epileptiform discharges. For our current study, we included 141 of the 168 EEGs with seizures (27 EEGs were excluded because of lack of demographic, clinical, or EEG data). We also randomly selected 71 of the 457 EEGs without seizures. This enrichment of seizure cases for the present study was meant to focus efforts on comparing physicians’ accuracy for detecting seizures when reviewing reduced versus full EEGs. We summarized demographic and clinical information for each patient, including age, gender, presence of intracranial hemorrhage, inpatient location (intensive care unit [ICU] or non-ICU), Glasgow Coma Scale (GCS), presence of coma (defined as GCS ≤ 8; e.g., absence of eye opening or verbal response to voice/pain, and inability to follow commands), and history of epilepsy.

### Phase I: Sensitivity and Specificity of rm-EEG with Asymmetric Access to Ancillary Information

Each continuous EEG recording (raw EEG data) was reviewed in its entirety alongside relevant auxiliary information (EEG spectrogram, video, clinical information, and the EEG report published in the medical record) by two neurologists at MGH during their epilepsy fellowship. Each reviewer categorized the EEGs as “seizure” (containing at least one seizure) or “non-seizure,” and their consensus was used as the fm-EEG diagnosis. Reviewers recorded the timing of the first electrographic seizure, and EEG samples were categorized into those with seizures within the first 4 h (*n* = 117), those with seizures after the first 4 h (*n* = 24), and those without seizures (*n* = 71).

We then digitally reduced each EEG to a montage consisting of ten electrodes covering the lateral circumference of the head (including the temporal chains, but excluding the parasagittal and midline chains; see Fig. 1C for an rm-EEG sample of burst suppression). The first 4 h of rm-EEG data (without spectral, video, or clinical information) were uploaded to the Ceribell cloud portal, and each rm-EEG was reviewed by one of five epilepsy fellows, who were asked to determine whether the EEG contained seizures, periodic patterns (generalized or lateralized periodic discharges [GPDs or LPDs]), epileptiform discharges (spikes or sharp waves), burst suppression, or slow/normal activity predefined according to the 2012 Standardized Critical Care EEG Terminology established by the American Clinical Neurophysiology Society [21]. To simulate clinical practice and provide an ecologically valid review process, the EEG was then reviewed by an epilepsy attending (author JP) who approved or rejected the fellow’s EEG interpretation. Although the attending was not blinded to the fellow’s interpretation, both the attending and the fellow were blinded to the fm-EEG diagnosis. While the attending read all the EEGs, each fellow was responsible for reading a subset. The numbers of EEGs read by each fellow (F) were as follows—F1: 60; F2: 25; F3: 89; F4: 21; F5: 44. If the attending and the fellow had the same interpretation, then this interpretation was used as the rm-EEG diagnosis. If there was disagreement, then a second epilepsy attending (author LJH) reviewed the EEG (blinded to *both* fm-EEG and rm-EEG diagnoses), and his decision—i.e., a majority consensus (2 out of 3)—was used as the rm-EEG diagnosis. We then compared the fm-EEG and rm-EEG diagnoses of seizure or non-seizure within the first 4 h to quantify the degree of concordance between fm-EEG with ancillary information and rm-EEG without ancillary information, both evaluated by a consensus of multiple reviewers, for seizure detection.

### Phase II: Sensitivity and Specificity of rm-EEG with Symmetric Access to Ancillary Information

In Phase I of the study, the rm-EEGs (4 h long) were rated as containing seizures, periodic patterns, epileptiform discharges, burst suppression, or slow/normal activity, whereas the fm-EEGs (> 18 h long) were classified more broadly into either seizure or non-seizure. Both of these categories may have included entities on the ictal–inter-ictal continuum (e.g., rhythmic or periodic patterns that could be ictal, but did not reach criteria for definite seizures). As noted previously, fm-EEGs and rm-EEGs were evaluated by different raters who had access to different levels of information. Full EEG readers had access to video, trending data, reason for EEG request (e.g., witnessed clinical seizures), medications, and a brief history of present illness, in addition to the EEG report available in the medical records. Moreover, the readers of fm-EEGs discussed the findings among themselves to come to a diagnostic conclusion. Fellows reading the rm-EEGs did so independently, without consulting with the attending, and without this additional information that might have caused fm-EEG readers to classify a periodic pattern as either seizure or non-seizure. We next sought to clarify whether discordant cases in Phase I were reflections of the *classification problem* or of the *detection problem*, and we sought to establish a “clean gold standard,” rooted entirely in EEG characteristics and free of external clinical contextual information, against which to judge the diagnostic value of rm-EEG. Toward this aim, we measured (1) intra-rater agreement between fm-EEG and rm-EEG for each individual reader by comparing how the individual’s reading of fm-EEG differed from the same individual reader’s interpretation of the same EEG in rm-EEG configuration and (2) sensitivity and specificity of rm-EEG versus fm-EEG when majority consensus was used as the gold standard.

For Phase II, we focused on EEG cases in which there was a discrepancy between fm-EEG and rm-EEG diagnoses in Phase I. For each of these EEGs, a 15-s EEG epoch was selected by one of the two original fm-EEG readers (MBW) from where the original fm-EEG readers had made their diagnosis. The 15-s epochs of fm-EEG and rm-EEG were shuffled and presented in random order to each of the five fellows and one epilepsy attending (author LJH), all of whom were blind to the EEG diagnosis. Each provided their diagnostic opinion as to whether an EEG contained seizures, periodic patterns, epileptiform discharges, burst suppression, or slow/normal activity. We used majority consensus (3 out of 5) as the final diagnosis, and an expert epileptologist’s responses (LJH) as a tiebreaker when needed. We did not have technical or financial means to ask the five EEG readers to review either the first 4 h or the entire record of fm-EEG data remotely.

### Association of Early rm-EEG Findings with Subsequent Seizures

After demonstrating the sensitivity and specificity of rm-EEG for various patterns, we next evaluated the association between periodic discharge patterns detected within the first 4 h of rm-EEG and the detection of seizures during the remainder of the EEG recording among the 95 EEGs without seizures in the initial 4 h. For this analysis, we used the original Phase I categorization of EEGs as seizure or non-seizure after 4 h based on review of fm-EEG as well as clinical and video information.

### Statistical Analysis

Specific EEG findings from Phase I and Phase II were classified as overt seizures, epileptiform patterns (including periodic discharges or epileptiform discharges), burst suppression, or slow/normal activity; these findings were further grouped as seizure (i.e., seizures only) and non-seizure (i.e., periodic discharges or epileptiform discharges, burst suppression, and slow/normal activity) for statistical analysis. Descriptive statistics (i.e., number and percentage, mean ± SD, and median [IQR]) were calculated for demographic and clinical characteristics. To describe the study population, differences in demographic/clinical factors between patients whose EEGs exhibited seizure activity versus non-seizure activity were summarized using one-way ANOVA, Kruskal–Wallis, and Chi-squared test statistics as appropriate. We used a significance threshold of *α* = 0.05 with Bonferroni’s correction for multiple comparisons.

We calculated the concordance between fm-EEG and rm-EEG in Phase I and identified the discordant cases for review in Phase II; here, concordance was defined as the percentage of cases in which classification of cases as seizure versus non-seizure was the same for fm-EEG and rm-EEG. For Phase II, we calculated intra-rater agreement between fm-EEG and rm-EEG, measured by the percentage of samples assigned the same label on both EEG montages by each rater, averaged across raters, and by Fleiss’ *κ*. We also calculated inter-rater agreement using fm-EEG and rm-EEG, measured by the percentage of samples assigned the same label on either rm-EEG or fm-EEG, averaged across pairs of readers, and by Fleiss’ *κ*. For Phase II analysis, we also calculated the sensitivity and specificity of rm-EEG for seizures relative to fm-EEG using majority consensus diagnoses for both fm-EEG and rm-EEG obtained in the Phase II survey.

We evaluated the predictive value of epileptiform activity (periodic discharges and epileptiform spikes) within the first 4 h of rm-EEG for subsequent seizure activity beyond the first 4 h, among patients who had not already had seizures within the first 4 h. We performed univariate, followed by multivariate, logistic regressions to calculate crude and adjusted odds ratios (with 95% confidence interval [CI]) of future seizures with epileptiform abnormalities and clinical variables (e.g., demographics and clinical history, ICU admission, and coma).

## Results

**Fig. 2:**
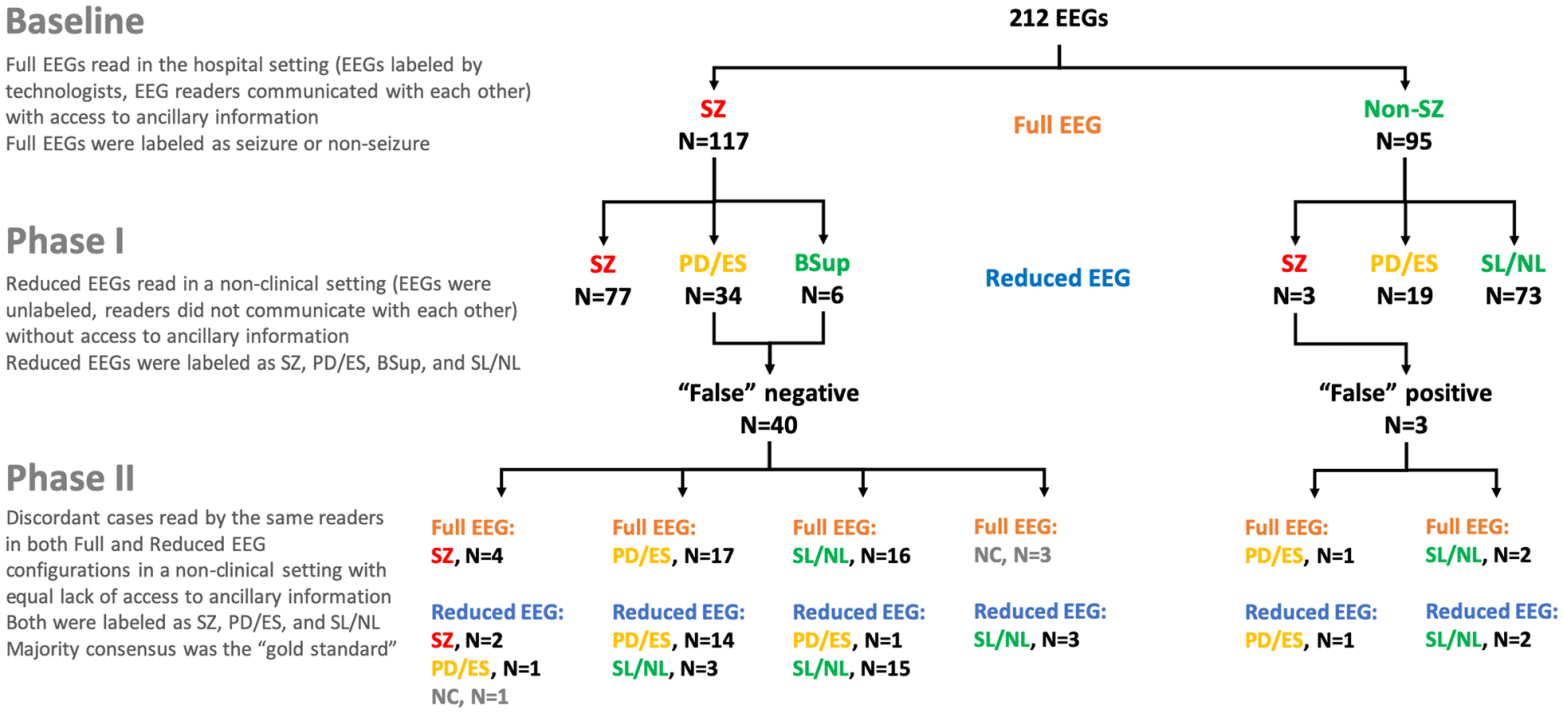
Flowchart of findings from Phase I and II. *BSup* burst suppression, *EEG* electroencephalography, *NC* no consensus, *PD/ES* periodic discharges or epileptiform spikes, *SL/NL* slow or normal activity, *SZ* seizure. Of note, 6 cases of burst suppression from Phase II were rated in fm-EEG review as either PD/ES (*N* = 2), SL/NL (*N* = 2), or reached no consensus (*N* = 2)

Demographic and clinical characteristics of our study population are given in Table 1. Most patients (86%) were admitted to the ICU, and over a third of patients (35%) had GCS ≤ 8. Compared to patients who ultimately did not have seizures, a greater percentage of patients with electrographic seizure activity (at any time) were admitted to an ICU (95% vs. 69%, *p *< 0.001) and were comatose (41% vs. 21%, *p *= 0.005). A flowchart of the findings from Phase I and Phase II is shown in Fig. 2.

**Table 1:** Patient characteristics and reduced EEG findings of 212 patients who underwent video EEG monitoring at Massachusetts General Hospital

Diagnosis of 18+ h of full EEG with access to video and trending data				
	Seizure within 4 h *N* = 117	Seizure after 4 h *N* = 24	Non-seizure *N* = 71	*p* value
Demographic and clinical characteristics				
Age, mean ± SD	65.6 ± 16.1	63.6 ± 18.8	62.6 ± 18.7	0.24*
Male gender, % (*n*)	53.8 (63)	41.7 (10)	38.0 (27)	0.092^†^
History of epilepsy, % (*n*)	29.1 (34)	16.7 (4)	16.9 (12)	0.11^†^
Intracranial hemorrhage, % (*n*)	25.6 (30)	33.3 (8)	36.6 (26)	0.27^†^
Admitted to ICU, % (*n*)	94.9 (111)	95.8 (23)	69.0 (49)	**<** **0.001**^†^
GCS, median [IQR] (range)	10 [11] (3–15)	11.5 [11] (3–15)	13 [5] (3–15)	0.081^‡^
Coma (GCS ≤ 8), % (*n*)	41.0 (48)	45.8 (11)	21.1 (15)	**0.010**^†^
Diagnosis of first 4 *h of reduced EEG*				
Seizure or epileptiform activity, % (*n*)	94.9 (111)	62.5 (15)	9.2 (7)	**<** **0.001**^†^
Seizure	65.8 (77)	12.5 (3)	0 (0)	
Periodic patterns	23.1 (27)	41.7 (10)	8.5 (6)	
Epileptiform spikes	6.0 (7)	8.3 (2)	1.4 (1)	
Non-epileptiform activity, % (*n*)	5.1 (6)	37.5 (9)	90.1 (64)	
Burst suppression	5.1 (6)	12.5 (3)	0.0 (0)	
Diffuse slow or normal activity	0.0 (0)	25.0 (6)	90.1 (64)	

*EEG* electroencephalography, *GCS* Glasgow Coma Scale, *ICU* intensive care unit

*p* values are calculated using one-way ANOVA (indicated by *), Chi-squared (indicated by ^†^) and Kruskal–Wallis (indicated by ^‡^) tests as appropriate; bolded *p* values are statistically significant

### Phase I: Concordance Between fm-EEG and rm-EEG Reviewed by Different Readers with Asymmetric Information

Of the 117 EEGs determined to have seizures within the first 4 h based on review of fm-EEGs recordings, 77 were labeled as seizures or status epilepticus, 27 with periodic discharges, 7 with sporadic epileptiform discharges, and 6 cases as burst suppression. None of the 117 cases with seizures on fm-EEG were labeled as non-epileptiform activity on the rm-EEG. Among the 95 cases not found to have seizure activity within the first 4 h on fm-EEG, raters of rm-EEG labeled 92 cases (97%) as non-seizure and 3 (3%) as seizures.

### Phase II: Concordance Between fm-EEG and rm-EEG When Majority Consensus was Used as Gold Standard and When fm-EEG and rm-EEGs were Reviewed by the Same Readers Without Access to Ancillary Information

**Fig. 3:**
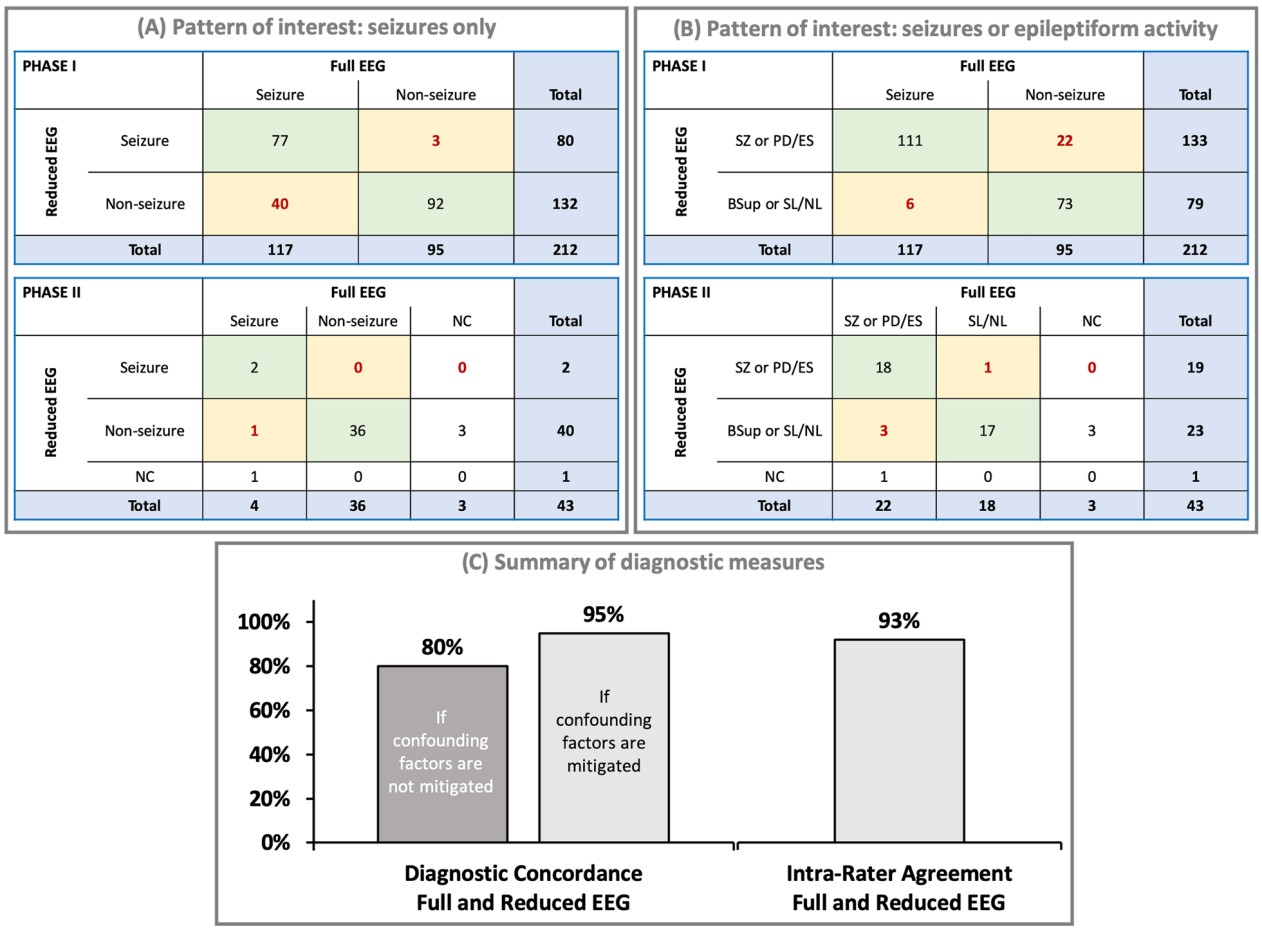
Agreement between fm-EEG and rm-EEG majority consensus diagnoses during initial 4 h of monitoring. Phase I (top) and Phase II (bottom) diagnostic tables using either seizures as the sole pattern of interest (**A**) or both seizures and epileptiform discharges as patterns of interest (**B**). Concordant cases indicated by green cells; discordant cases indicated by yellow cells with bolded red text. Diagnostic concordance and intra-rater agreement are shown in (**C**). *BSup* burst suppression, *NC* no consensus, *PD/ES* periodic discharges or epileptiform spikes, *SL/NL* slow or normal activity, *SZ* seizure

The results of Phase II are summarized in Figs. 2 and 3. As noted, of 117 fm-EEGs with seizures within the first 4 h, 40 were labeled by reviewers of rm-EEG as having no seizures (seemingly *false negative* cases, although none was categorized as only slow/normal), while 3 of the 24 fm-EEGs without seizures within 4 h were labeled as having seizures on rm-EEG (seemingly *false positive* cases) (Fig. 3A, top). This brought the total number of discordant samples among EEGs with seizures at any time to 43 (30.5%). We asked the same five fellows and one attending, all of whom were blinded to the findings of Phase I, to review all 43 discordant EEGs in *both* fm-EEG and rm-EEG formats (presented randomly). It should be noted that rm-EEGs in Phase I were read by two to three readers (depending on whether the first two readers’ diagnoses agreed) who had not evaluated the fm-EEG, whereas both the fm-EEG and rm-EEG in Phase II were read by six readers (using majority consensus as the gold standard).

**Fig. 4:**
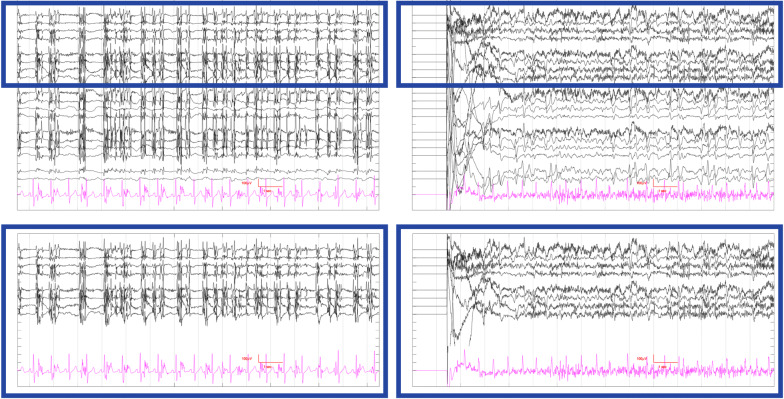
Samples of seizure activity diagnosed on fm-EEG, but classified as non-seizure on rm-EEG. Reduced EEG channels indicated by the blue box. The first sample contained generalized activity that was classified as seizure by the majority of reviewers using fm-EEG (top left); no majority consensus was achieved using rm-EEG (bottom left); however, the expert epileptologist diagnosed this activity as seizure using both fm-EEG and rm-EEG. The second fm-EEG sample (top right) shows focal parasagittal seizure activity that is not visible on rm-EEG (bottom right) and was interpreted as epileptiform spikes by the majority of reviewers. EEGs are shown in the fm- and rm-configurations shown in Fig. 1. EKG shown in pink

Among the 40 presumed false negative cases (labeled as seizure within the first 4 h on fm-EEG and non-seizure on rm-EEG in Phase I), Phase II analysis showed only four (10.0%) were labeled as seizure on fm-EEG, and two of these were also labeled as seizure on rm-EEG (Fig. 3A, bottom). The remainder were classified as showing epileptiform discharges (42.5%), burst suppression (5.0%), or slow/normal activity (35.0%). We reviewed both burst suppression cases (see Fig. 1C for a representative example) and found that they were predominantly suppressed during the first 4 h. The three presumed false positive cases (labeled as non-seizure within the first 4 h on fm-EEG and seizure on rm-EEG in Phase I) were all reclassified as non-seizure cases on both fm-EEG and rm-EEG in Phase II. Figure 4 shows the two cases that were labeled as seizure on fm-EEG, but were missed in the rm-EEG in Phase II.

Three false negative samples (7.5%) did not generate a majority consensus on fm-EEG in Phase II, all of which were labeled as non-seizure on rm-EEG. Only one false negative sample (2.5%) did not generate a majority consensus on rm-EEG; however, this sample was labeled as seizure on fm-EEG. Individual rater diagnoses of these samples without consensus on either fm-EEG or rm-EEG are given in Table 2, which reflects that, although raters did not agree on their specific diagnosis, their impressions did mostly agree on whether a sample was seizure-like or non-seizure.

**Table 2:** Samples that did not have a majority consensus on either fm-EEG or rm-EEG in Phase II

Sample	Montage	Rater 1	Rater 2	Rater 3	Rater 4	Rater 5	Rater 6^a^	Majority
No consensus diagnosis on fm-*EEG*								
1	fm-EEG	SL/NL	PD/ES	SZ	PD/ES	SZ	BSup	NC
	rm-EEG	BSup	BSup	SZ	PD/ES	SZ	BSup	BSup
2	fm-EEG	SL/NL	SZ	PD/ES	PD/ES	SZ	SL/NL	NC
	rm-EEG	SL/NL	SL/NL	PD/ES	SL/NL	SL/NL	SL/NL	SL/NL
3	fm-EEG	SZ	SL/NL	PD/ES	SL/NL	SZ	PD/ES	NC
	rm-EEG	SL/NL	SL/NL	PD/ES	PD/ES	SL/NL	PD/ES	SL/NL
No consensus diagnosis on rm-*EEG*								
4	fm-EEG	SZ	SZ	SL/NL	PD/ES	SZ	SZ	SZ
	rm-EEG	PD/ES	BSup	SL/NL	SL/NL	SZ	SZ	NC

*BSup* burst suppression, *fm-EEG* full montage EEG, *NC* no consensus, *PD/ES* periodic discharges or epileptiform spikes, *rm-EEG* reduced montage EEG, *SL/NL* slow or normal activity, *SZ* seizure

^a^Rater 6 served as a tie‐breaker when no majority diagnosis was reached between raters 1 through 5

The concordance between fm-EEG and rm-EEG observed in Phase II (excluding samples that did not generate a majority consensus on fm-EEG) was 95% (Fig. 3A). The inter-rater agreement among the six readers for all 43 discordant cases (measured with Fleiss’ *κ*) was 0.37 for fm-EEG and 0.25 for rm-EEG, indicating only fair agreement between raters even though each rater had access to the same amount of information. This suggests that these cases may have been intrinsically difficult to categorize. We observed higher intra-rater agreement (concordance: 93.4 ± 4.3%; average within-rater Fleiss’ *κ* per rater: 0.45 ± 0.18, range: 0.42–0.88) (Fig. 3C).

After reclassifying the discordant samples according to the results of Phase II and combining these with the concordant samples from Phase I, the overall concordance between fm-EEG and rm-EEG increased from 79.7 to 99.0%, and the sensitivity and specificity of rm-EEG compared to fm-EEG in detecting seizure cases increased to 97.5% and 100.0%, respectively.

We also analyzed Phase I and Phase II data using both seizures and epileptiform activity (periodic discharges and epileptiform spikes) as patterns of interest (Fig. 3B), as opposed to our prior analysis concerned with seizures alone. Reanalysis of Phase I data grouping all epileptic activity resulted in a reduction in “false negative” cases and an increase in “false positive” cases (Fig. 3B), resulting from reclassification of 53 samples diagnosed as periodic discharges or epileptiform spikes on rm-EEG (34 previously “false negative” cases now considered concordant cases and 19 previously concordant cases now reclassified as “false positive” cases). It should be noted that interpretation of this Phase I reanalysis is limited by the fact that reviewers of fm-EEG in Phase I did not indicate the presence of non-seizure epileptiform activity within the first 4 h. However, Phase II reanalysis (Fig. 3B) demonstrates that rm-EEG displayed high concordance (87.5%), sensitivity (81.8%), and specificity (94.4%) in detecting epileptic activity in general.

### Association of Early rm-EEG Findings with Subsequent Seizures on fm-EEG

We also explored whether early information obtained with rm-EEG could predict seizures detected with subsequent fm-EEG monitoring. For this, we compiled pathological findings in the first 4 h of rm-EEG in those 95 patients who did not have seizures detected in the first 4 h of conventional EEG monitoring. We then excluded the 3 patients who were diagnosed with seizures on rm-EEG, leaving 92 patients who did not have seizures on either fm-EEG or rm-EEG in the first 4 h of monitoring for subsequent analysis. As given in Table 3, 12 of the 19 patients (63.2%) diagnosed with epileptiform activity on rm-EEG during the first 4 h went on to have seizures on subsequent long-term monitoring, whereas only 9 of the 73 patients (12.3%) without epileptiform abnormalities in the first 4 h of rm-EEG had seizures captured with fm-EEG in subsequent days. Moreover, the presence of epileptiform patterns [e.g., GPD, LPD, or spikes] in the first 4 h of rm-EEG recording was associated with significantly increased odds of detecting future seizures (OR 12.19, 95% CI 3.94–41.37, *p *< 0.001). ICU admission (OR 9.98, 95% CI 1.70–166.16, *p *= 0.038) and coma (OR 4.12, 95% CI 1.48–11.74, *p *= 0.007) also increased the odds of detecting a seizure after 4 h of monitoring. However, when these variables were included in a multivariate logistic regression model, the presence of periodic discharges or epileptiform spikes was the only significant predictor of future seizures (OR 8.81, 95% CI 2.56–34.28, *p *< 0.001).

**Table 3:** Risk of future seizures associated with epileptiform patterns within 4 h of reduced EEG and demographic and clinical characteristics

	Future seizures, % (*n*)	Crude OR (95% CI)	*p* value	Adjusted OR (95% CI)	*p* value
Age ≥ 65 years	22.0 (11)	0.90 (0.34–2.43)	0.84	1.22 (0.32–4.92)	0.77
Male gender	27.0 (10)	1.48 (0.55–3.98)	0.43	2.52 (0.68–9.80)	0.17
History of epilepsy	14.3 (2)	0.52 (0.08–2.13)	0.42	0.37 (0.04–2.21)	0.32
Intracranial hemorrhage	21.2 (7)	0.87 (0.30–2.37)	0.78	0.61 (0.14–2.59)	0.51
ICU admission	29.0 (20)	8.98 (1.70–166.16)	**0.038**	4.87 (0.60–106.15)	0.19
Coma	42.3 (11)	4.11 (1.48–11.74)	**0.007**	2.49 (0.72–9.08)	0.15
Any epileptiform activity^a^	63.2 (12)	12.19 (3.94–41.37)	**<** **0.001**	8.81 (2.56–34.28)	**<** **0.001**

Logistic odds ratios are presented with 95% confidence intervals (CI) obtained from univariate (crude) and multivariate (adjusted) regression

*EEG* electroencephalography, *ICU* intensive care unit, *OR* odds ratio

Bolded *p* values are statistically significant (*α* = 0.05)

^a^Epileptiform abnormalities included periodic patterns (generalized and lateralized periodic discharges) and epileptiform spikes, while slow or normal activity (including burst suppression) were considered non-epileptiform activity

## Discussion

Our retrospective study of 212 EEGs demonstrates that rm-EEG, when reviewed without trending and clinical information (Phase I), as compared with fm-EEG reviewed with full access to spectral trending, video information, and patient’s clinical history, displays excellent concordance (95% agreement) and positive predictive value (96%) regarding the presence of pathological epileptiform activity (seizures, periodic discharges, or epileptiform discharges), but only reasonable concordance (73%) regarding the type of pathologic epileptiform activity (e.g., status epilepticus versus GPDs). Where discrepancies were noted between fm-EEG and rm-EEG diagnoses in Phase I, we found a high degree of intra-rater agreement (93%) between fm-EEG and rm-EEG (Phase II). These findings, taken together, suggest that the discordance between full and reduced EEGs in Phase I regarding the type of epileptiform activity was mostly due to inter-rater variability in classification and differences in access to clinical, video, and quantitative trending information (which were available to readers of fm-EEG and not to readers of rm-EEG), rather than a true difference in detection sensitivity caused by the reduction in the number of EEG channels.

Recent literature, including our own, suggests that EEG findings detected in brief recordings can predict the risk of seizures detected in long-term recordings in critically ill patients [26, 27, 33–35]. These studies were performed using fm-EEG, and before the present study, it was theoretically possible that rm-EEG might not preserve this previously described predictive value. Inspired by the extant evidence, we looked at the association between pathological findings in the first 4 h of rm-EEG and seizures in the subsequent hours of monitoring. Our findings demonstrate that detection of periodic discharges or epileptiform spikes in the first 4 h on rm-EEG is associated with increased odds of future seizures (12/9, or 133%), as much as twelve times more than if only slow or normal activity was detected (7/64, or 11%).

Stat EEG has traditionally been performed using the International 10-20 system, the same electrode array used for non-urgent outpatient diagnostics. While this array provides greater spatial coverage, this comes at a cost (e.g., electrodes, technologist time and experience to apply electrodes, and time to signal acquisition) and may result in significant delays in diagnosis [36, 37]. The value of rm-EEG lies in its ability to enable emergent evaluation of critically ill patients suspected to have subclinical or non-convulsive seizures, for whom such delays in diagnosis and treatment impact morbidity and mortality [38]. Diagnostic assessments of these patients must be obtained quickly, and the EEG data obtained should be reliable enough to direct management and triage patients effectively into high-risk or low-risk categories. Indeed, prior studies have demonstrated that specific reduced electrode arrays are more rapidly deployed at the bedside by clinical personnel without requiring the training of EEG technologists compared to fm-EEG [10, 39]. In addition, a prior report [30] that utilized 15-s EEG samples and the present study involving 4 h of continuous monitoring demonstrate that a ten-electrode rm-EEG montage has both excellent sensitivity for detecting generalized or hemispheric seizures that typically warrant intervention, and excellent specificity for discriminating more benign slow or normal activity from pathologic patterns that can identify patients at higher risk of seizures [40]. Overall, the diagnostic and predictive utility that rm-EEG offers in urgent and critical care settings may be valuable for directing acute management with antiepileptic drugs, as well as triaging conventional EEG monitoring resources toward those patients at highest risk for seizures.

## Conclusions

Our findings demonstrate that differences between fm-EEG and rm-EEG observed in prior studies, as well as in the first phase of this study, can largely be explained by variability in EEG pattern classification across readers and incorporation of asymmetric clinical information into EEG interpretation rather than by true detection failure of rm-EEG as a result of electrode reduction. EEG with circumferential configuration with ten electrodes preserves key features of the traditional EEG system, namely its diagnostic utility for detecting seizures and periodic discharges, and presents a useful triage method for identifying high-risk patients.

## Data Availability

Summary data are given in Tables 1, 2, and 3. Patients’ raw EEG data are protected by HIPAA regulations. Any other related documents can be shared upon request from the corresponding author.
